# Case of Pseudo-Membranous Laryngitis, (Croup,) Treated by Topical Application of the Nitrate of Silver

**Published:** 1850-06

**Authors:** P. H. Strong

**Affiliations:** Buffalo


BUFFALO, 1850.

---

*Case of Pseudo-membranous laryngitis, (croup,) treated by topical application of the nitrate of silver.* BY P. H. STRONG, M. D.

DR. FLINT,— DEAR SIR:

Since you are pleased to regard the following case as of sufficient interest to the profession to merit publication, I do not feel at liberty to decline your request to that end. Herewith you have my notes of the case, mainly as they were jotted down at the time, with little thought of publication.

Wednesday evening, 9½ o'clock, April 10, 1850, I was summoned to see a child of Mrs. M——, North Division street, but resident of Chase county, and just from there. Found her a fair, robust child, of 3½ years, laboring under well marked symptoms of fully developed croup. Pulse rapid, skin burning, slightly livid, respiration frequent, laborious, markedly stridulous, with head thrown back, nostrils compressed, cough characteristically dry and ringing, though perhaps more suffocated and bronchial than in usual form, voice reduced to a whisper. They dated the first of croupal symptoms some five hours anterior to my visit, from exposure to chilling wind,—though she had not appeared quite well, I believe, for some days previous. Having neither of my favorite emetics for such cases at hand, (*Blood root* and *Turpeth mineral,*) and the necessity for nausea and emetics being urgent, I gave her Ipecac. and tartar emet. for that purpose. After a thorough vomiting she seemed sensibly relieved in respiration, cough, heat of skin, etc. I left her for the night, upon two grs. of calomel every two hours, with a contingent *Turpeth mineral* emetic upon the least aggravation of symptoms. They thought the emetic indicated in the middle of the night, and gave it with evident relief.

Thursday morning, April 11.—At my visit she seemed decidedly better, with croupal symptoms but trifling; continued calomel every four hours, and directed nauseant and expectorant doses of Inf. Sanguinaria statedly, and sufficient of same to vomit upon re-appearance of symptoms, and if she grew worse, to summon me at once.

Friday, April 12.—At my stated visit this morning, there was a marked aggravation of symptoms, in rapidity of pulse, heat and hue of skin, frequent and stridulous respirations, cough, etc., etc. They now reported her as getting worse through the night, but for some unaccountable reason they had failed to apprise me of it.

As the calomel was loosening the bowels undesirably, and the emetic failed of full relief, I felt anxious to try the topical treatment by strong *Nit. Arg.*

Having had neither experience in, nor observation of the treatment, I hastily consulted several medical gentlemen, who were alike inexperienced. Dr. White, having had the nearest to an experience, of any one my time allowed me to see, his advice and assistance were requested, and obligingly rendered.

The bent probang, armed with a fine sponge of some ⅝ inch diameter when dry, and saturated in solution of crystals of *Nit. Arg.*, ℈ii to an oz. of water, was applied to the *fauces* and *epiglottis,* and thence passed into the *aperture of the glottis,* without difficulty, and with little distress to the patient. The sponge, when returned, was somewhat coated with tenacious mucus, and the patient vomited slightly of the same immediately. The effect upon the symptoms was not very marked; the stridulous respiration was, perhaps, slightly lessened. Not being fully satisfied, though encouraged by this trial, we thought it advisable to meet on the case again in 1½ hours, and repeat it if indicated. Accordingly we met again at 11½ A. M., and deeming the topical treatment as the only hope for the little sufferer, and there being, at least, no important results from the former trial, we repeated the application, with more than double the former strength, (℈iv to i℥). The sponge now was more freely coated with *muco-fibrinous* secretion, and more was again ejected by vomiting, with shreds of *albuminous exudation.* The disturbance to the patient by strangling was but slight and momentary, and the relief to the breathing was more palpable than before—though not all we could wish. We concluded to meet again at 3½ o'clock, P. M., and watch the result. At that hour the difficulty in respiration seemed not so referable to the *Rima-glottidis,* as to lower points in the air passages. The characteristic croupal symptoms might be said to be relieved somewhat, but the energies of the system seemed flagging—it being now some forty-eight hours from the onset of the disease. We viewed the case as less hopeful; but the caustic application suggested to our mind, the *only* hope, and a re-application of the same was tried, with but partial relief. Patient continued through the day and succeeding night, with no change, except a gradual sinking of vital energies, till Saturday, April 13, 9 o'clock, a. m., at which time her symptoms presented in this wise: Pulse very weak and so rapid as not to be counted; respirations thrice their normal frequency, suffocative and stridulous, though this apparently was more traceable to the *larynx* or *trachea* than to the *glottis*. Again the caustic application appeared the only hope, and a forlorn one at that. Promising not to lessen its chance, it was again resorted to, with little relief resulting for the time. Indeed for some five or six hours thereafter, the prognosis seemed so unpromising as almost to forbid persistance in the attempt to administer any thing. For that length of time a fatal termination was hourly looked for. I however did persist in recommending a preparation of *sanguinaria* and *lobelia* while she could swallow. She continued apparently failing till night-fall, when she fell asleep, and continued to rest well through the night, her oppressive and stridulous respiration abating hour by hour. Such was their morning report of her.

You, perhaps, Mr. Editor, may imagine, I cannot express, the nature and extent of my surprise, when, at my 9 o'clock morning visit the next day, Sunday 14th, I found my patient neither dead nor dying, as was my expectation, but alive and with a better prospect for continuing so, than she had had since the date of her attack, some three and a half days previous. All her urgent symptoms were effectually mitigated—croupal cough and respiration, cyanosed hue of skin, ebrile excitement, etc. She continued to improve steadily from that time forward to complete restoration to health.

In taking a retrospect of the case, while I would not deny qualified power to the other means resorted to, my conviction was, and is, that the case was hastening to a fatal result, and that not long deferred, till recourse was had to the topical treatment by caustic.

And whilst I would not underrate the inherent recuperative powers of the system, and the *possibility* of an agreeable termination in this case without it, and aware moreover of the evil of deducing too rash conclusions from single cases of results of treatment, still the marked effects that followed the application, as the case presented, suggests it very clearly to my mind as saving the life of my patient. I am happy in this to have the concurring opinion of the counsel in the case.

One or two points seem worthy of particular notice:

1st. The surprising impunity with which so strong (iv. to v. drach. to the ℥.) a solution of nitrate silver (stronger so far as I know, than Dr. Green or any other advocate of the practice has recommended), was passed through the rima-glottidis and freely applied to the inner surface of the larynx, and that, too, without appreciable inconvenience to the little patient, except at the moment of obstruction of the passage by the sponge.

2nd. The varying effects of the application at the different stages. When the characteristic respiration and cough were plainly referable to obstruction quite at the opening of the larynx, by the adventitious membrane, the relief was immediate and palpable. Later in the disease, when the obstruction seemed lower in the air passages, the relief was not so evident *at once*, but most gratefully so after the lapse of a few hours. This may be accounted for from the intimate nervous sympathy existing between different points of the same membrane—or, as Doctor Green supposes, from the actual contact of the caustic liquid, as it courses it way down the mucous surface. The sponge being saturated, and thus freely imparting its contents upon touching any point, this last opinion seems not irrational.

This case is the more readily furnished you, Mr. Editor, from the conviction with which the present reporter is possessed, that my professional brethren (here at least,) have hitherto failed to consider (as the undersigned confesses to have done) the claim that this practice has upon our regard. This may have obtained measurably from the fact that this locality has hitherto been far less obnoxious to that terrific disease *Croup*, than more easterly localities. However this may be, it now evidently has become of sufficient frequency, and stalks through its course sufficiently defiant of our ordinary means, especially when summoned, (as is usually the case perhaps) only after the disease is fully formed, as to make it excusable, if not imperative, to consider well a more excellent way—if such is commended to us by facts well attested—the results of actual, cautious experience.

Is it said, or oftener thought, "The practice is quite too heroic?" It may be replied, with equal truth, that we have to do with an heroic enemy, And the skillful general is made such, precisely by considering well the position and strength of his foe, and mustering and marshaling his resources accordingly. An advanced stage of a case of croup is eminently a crisis in which, what is well done, must needs be done boldly and quickly. No one can have felt more distrustful of the recommendation, when first broached, of freely introducing a concentrated solution of lunar-caustic directly into the larynx of a child, nor more shrinking and fearful at my first trial of it, than the present writer. Still, I could not shut my eyes, neither methinks can any one, to the array of evidence in its favor; my own being only an isolated case added to a multitude that are recorded.

I may be mistaken in the assumption that it is not practised by my professional brethren in this city. If so, my object in reporting this will be gained by securing, if possible, the report of such cases as may exist. And if not mistaken, my reward will be full, to have commended to the practical consideration, and to have attracted the attention of the profession to what appears an important resource in a real exigency.

I shall not soon forget my anxiety, when my patient was almost at death's door by suffocation, to find some medical friend who could say *he had tried it*, to relieve measurably that trepidation which we are apt to feel, at first trial of (to us) untried and bold expedient.

In the hope that the case will fulfil your expectations in subserving the interest of our profession, I subscribe myself,

Yours respectfully,

P. H. STRONG.

BUFFALO, April 1850.

